# Sperm-egg fusion disorder in a Chinese male patient was associated with a rare *ADAM20* variant

**DOI:** 10.18632/oncotarget.23331

**Published:** 2017-12-16

**Authors:** Yan-Wei Sha, Xiaohui Xu, Zhi-Yong Ji, Li-Bin Mei, Ping-Ping Qiu, Hong Ji, Ping Li, Lin Li, Wei-Wu Liu

**Affiliations:** ^1^ Department of Reproductive Medicine, Xiamen Maternity and Child Care Hospital, Xiamen, Fujian 361005, China; ^2^ School of Pharmaceutical Sciences, Xiamen University, Xiamen, Fujian 361005, China; ^3^ Central Laboratory, Beijing Obstetrics and Gynecology Hospital, Capital Medical University, Chaoyang, Beijing 100026, China; ^4^ Department of Radiology, the 2nd Hospital Affiliated to Jilin University

**Keywords:** Sperm-egg fusion, ADAM20, whole-exome sequencing, in vitro fertilization

## Abstract

We report here a 28-year-old male with infertility. No abnormality was found in his semen examination. The couple achieved a successful pregnancy under the help of intracytoplasmic sperm injection during which we found that sperm could enter the zona pellucida, but could not fuse with the egg within the short insemination period. We then performed whole-exome sequencing technology on this patient and found a rare variant (c.641A>C:p.D214A) in *ADAM20*, which encoded a disintegrin and metalloprotease 20 protein. To further verify the pathogenicity of this variant, we analyzed ADAM20 protein expression in spermatozoa by immunostaining analysis, which showed a mis-localization of ADAM20 in the patient's spermatozoa. Therefore, we concluded that mutation in *ADAM20* may be associated with sperm-egg fusion disorder in this patient.

## INTRODUCTION

Fertilization is a complex process involving many molecules. Sperm-egg fusion is one of the most critical events in sexual reproduction. The sperm is activated in the female reproductive tract after capacitation, resulting in modification of the membrane composition and enhancement of membrane fluidity, leading to sperm hyperactivation. Sperm will then reach the cumulus, pass through layers of cumulus cells, and bind to the zona pellucida (ZP), triggering the acrosome reaction. The acrosome reaction releases a large number of enzymes that hydrolyze the local ZP such that the sperm can pass through and enter the perivitelline space. The acrosome reaction also exposes the acrosome intima and alters the equatorial region and the membrane components of the post-acrosomal region [1]. Having reached the perivitelline space between the egg ZP and plasma membrane, sperm binds to and fuses with the plasma membrane at the equatorial region [2].

Binding of sperm to the egg plasma membrane is thought to be mediated by A Disintegrin-like And Metalloproteinase-containing proteins (ADAMs) [3], Izumo sperm-egg fusion proteins (IZUMOs) [4], cysteine-rich secretory proteins (CRISPs) [5], and mannose-ligand receptor [6]. ADAM proteins which anchored to the cell membrane, are a class of cell surface proteins that contain four conserved domains: the proteolysis domain, adhesion domain, fusion domain, and intracellular signal domain. These proteins are sperm membrane proteins expressed in the testes and are thought to play important roles in sperm-egg binding and sperm-egg membrane fusion [3]. The extracellular portion of ADAM1 in recombinant rats can be combined with the microvillar region of mouse oocytes and inhibit sperm-egg binding [7]. Human ADAM20 and ADAM1 have several common features [8], including the same metalloprotease activity and a conserved predicted fusion polypeptide active site. However, the only human *ADAM1* gene (also known as fertilin α) is non-functional [9]. Therefore, the questions about which gene play an important role in human sperm-egg fusion, and whether ADAM20 is involved in this process remain unclear. Until now, no study has reported the association of ADAM20 mutations with male infertility.

In this paper, we report a patient with sperm-egg fusion disorder harbored a heterozygous *ADAM20* rare variant. The localization of ADAM20 protein was presented as ring-structure and acrosome staining in the normal spermatozoa, while in the sperm of patient the ring-structure and acrosome staining of ADAM20 disappeared. As far as we know, this is the first study to report the genetic alteration of sperm-egg fusion disorder in human.

## RESULTS

### Patient with sperm-egg fusion disorder

**Figure 1:**
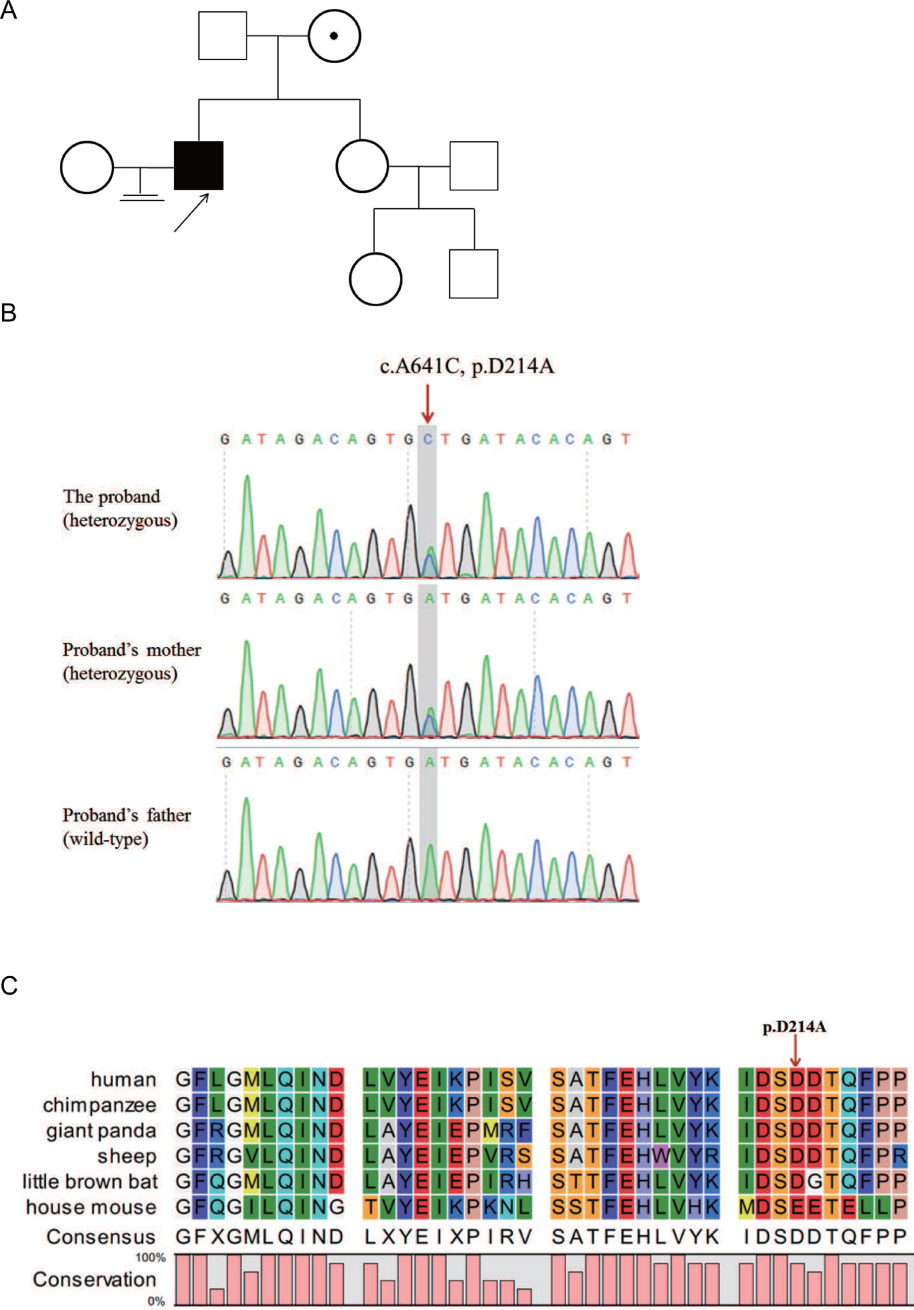
A patient with sperm-egg fusion disorder in a family (**A**) Family tree of the patient. The black arrow points to the proband. (**B**) Sanger sequencing confirmed the *ADAM20* variant in the proband. The proband carried a heterozygous *ADAM20* mutation (c.A641C). The patient's mother carried heterozygous allele, and his father harbored the wild-type sequence. The red arrow points to the variant site. (**C**) Alignment of ADAM20 protein in different species. The red arrow points to the D214 amino acid.

In our practice, we encountered a male patient (28 years of age, married in 2012) who had normal erection, ejaculation, and sexual activity (2–3 times per week). However, despite not using contraception, his wife had not become pregnant. His parents were not consanguineous, and his sister had given birth to two healthy babies (Figure 1A). The patient was employed in the field of computer maintenance and had no history of hazardous environmental exposure or poor habits such as smoking or drinking. Physical examination results were as follows: height, 168 cm; weight, 70 kg; external genital development, normal; and bilateral testicular size and bilateral spermatic vein, normal. Peripheral blood chromosomes showed no abnormalities, and no microdeletions were found in Y chromosome by G-banding and real-time PCR. Moreover, no abnormalities were observed on examination of semen at another hospital. The semen examination revealed a semen volume of 3.5 mL, sperm density of 125.8 million/mL, and sperm viability of 67.5%. Sperm morphology modified by Papanicolaou staining showed that 4% of sperm exhibited a normal morphology (Supplementary Figure 1A–1C). Additionally, sperm acrosin was 25 mIU/mL, and electron microscopy analysis revealed the presence of the sperm acrosome.

We collected twenty-four eggs (23 at the stage of two pronuclei and one at the germinal vesicle stage) after treating the patient's wife by *in vitro* fertilization (IVF) due to tubal factor. We found that sperm could enter the ZP, but could not fuse with the egg within the short insemination period (Supplementary Movie 1). Accordingly, we then considered rescue ICSI and performed a sperm-egg crossover experiment after obtaining permission from the interim ethics committee of our institution. The patient's sperm still could not fuse with a third-party egg by normal *in vitro* fertilization. Then the eggs of the patient's wife were normally fertilized using ICSI, yielding nine high-quality embryos (one was excellent) on the third day. In order to avoid excessive ovarian stimulation, two of the frozen embryos were transplanted on August 2016, resulting in a successful pregnancy.

### WES analysis of the patient

**Figure 2:**
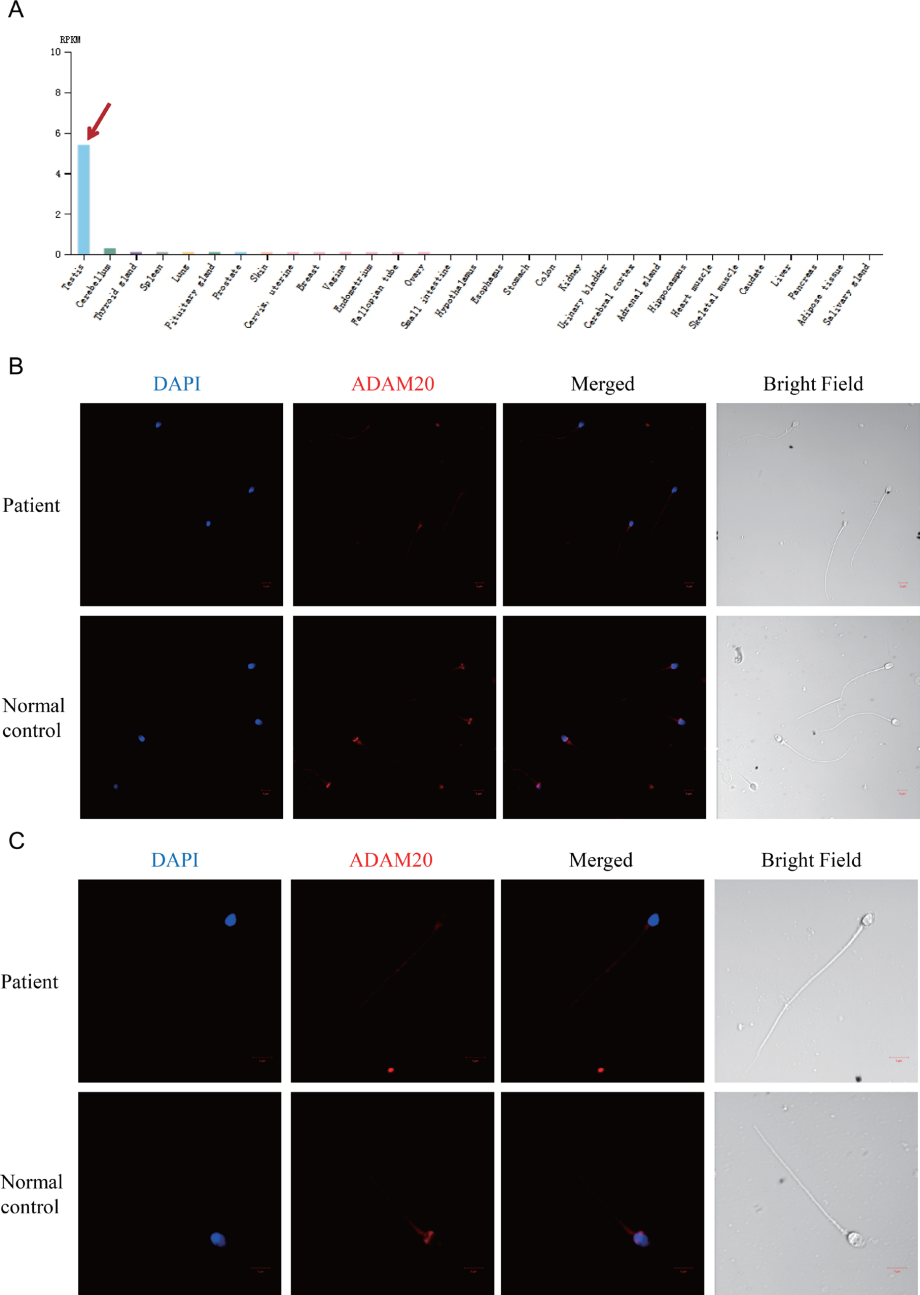
The expression pattern of ADAM20 changed in the patient's spermatozoa (**A**) *ADAM20* was highly and specifically expressed in human testis. The data was from the online database, http://www.proteinatlas.org/ENSG00000134007-ADAM20/tissue The red arrow points to the expression level of *ADAM20* in testis. (**B**) Immunostaining of ADAM20 in the sperm of both the patient and normal control. The ADAM20 protein was stained in red. (**C**) Enlarged pictures of immunostaining of ADAM20 in the sperm of both the patient and normal control.

Patient description analysis revealed the abnormality in the sperm. Thus, we focused on the rare variants in the proband identified by whole-exome sequencing. We filtered out polymorphisms with allele frequency greater than 0.1% in the dbSNP, 1000 Genomes, ESP6500siv2 and ExAC databases, and a list of genes harboring homozygous/heterozygous sequence variants were analyzed. Among these genes and rare variants, *ADAM20* is abundantly and specifically expressed in testis (Figure 2A). Importantly, ADAM20 belonged to ADAM protein family, several members of which are involved in binding to egg plasma membrane [3]. Therefore, we hypothesized that the rare variant in *ADAM20* was associated with sperm-egg fusion disorder. By means of Sanger sequencing, the heterozygous variant in *ADAM20* (NM_003814:exon2:c.641A>C:p.D214A), was confirmed in the patient (Figure 1B). The proband inherited this variant from his mother, while his unaffected father did not carry this rare variant.

### *In silico* analysis of the *ADAM20* variant

*In silico* analysis predicted that the *ADAM20* variant c.641A>C is possibly a deleterious mutation by PROVEAN (Table 1). This variant is a novel variant absent from the gnomAD and 1000 Genomes databases, respectively (Table 1), and it is also with extremely low allele frequency in ExAC database (Table 1), which is consistent with the rarity of this disorder. The variant site D214 was highly conserved from human to mouse, indicating an important role of this site (Figure 1C).

**Table 1:** *In silico* analysis of *ADAM20* mutation

Mutation	Amino acid change	Polyphen-2^a^	SIFT^b^	PROVEAN^c^	Mutation Taster^d^	SNPs&GO^e^	ExAC (total)^f^	ExAC (East Asian)^g^	1000 Genomes^h^	gnomAD^i^
c.A641C	p.D214A	Benign (0.014)	Tolerated (0.058)	Damaging (–3.59)	Polymorphism (0.9999)	Neutral (0.140)	0.00002035	0.0002899	0	0

^a^Polyphen-2 (http://genetics.bwh.harvard.edu/pph2/). Prediction Scores range from 0 to 1 with high scores indicating probably or possibly damaging.

^b^SIFT, i.e., Sorting Intolerant From Tolerant (http://sift.jcvi.org/). Scores vary between 0 and 1. Variants with scores close or equal to 0 are predicted to be damaging.

^c^PROVEAN (http://provean.jcvi.org/protein_batch_submit.php?species=human). Variants with scores lower than -2.5 (cutoff) are predicted to be deleterious.

^d^Mutation Taster (http://www.mutationtaster.org/). The probability value is the probability of the prediction, i.e., a value close to 1 indicates a high ‘security’ of the prediction.

^e^SNPs&GO (http://snps.biofold.org/snps-and-go/). Probability: Disease probability (if >0.5 mutation is predicted Disease).

^f^Frequency of variation in total of ExAC database.

^g^Frequency of variation in East Asian population of ExAC database.

^h^Frequency of variation in 1000 Genomes database.

^i^Frequency of variation in total of gnomAD (genome Aggregation Database, a big database containing 123,136 exome sequences and 15,496 whole-genome sequences).

### ADAM20 with D214A mutation mislocalized in the sperm head

To further verify the pathogenicity of the D214A variant, we analyzed ADAM20 protein expression in spermatozoa from the patient and control by immunostaining. ADAM20 protein was localized as ring-structure around the sperm head in normal control (Figure 2B and 2C). Besides, the acrosome region was also stained with relative weak signal of ADAM20 in control sperm (Figure 2C). However, in the patient, both the ring-structure and acrosome staining disappeared in the sperm head (Figure 2B and 2C). Therefore, the distribution of ADAM20 protein in the patient suggested that the D214A mutation might affect the localization of ADAM20.

## DISCUSSION

We report here a rare mutation, p.D214A, of the ADAM20 in a male patient with sperm-egg fusion disorder. The localization of mutated ADAM20 protein changed, with the ring-structure and acrosome staining disappearing. While this mutation appeared to affect sperm-egg binding and fusion, it did not affect later embryonic development or clinical pregnancy.

*ADAM20* was a testis-specific expressed gene [8]. ADAM20 was closely related to ADAM1 and ADAM2 [8]. Because of the human *ADAM1* gene is non-functional [9], it is speculated that ADAM20 may play the same role and substitute for ADAM1 in human sperm [8, 10]. Therefore, ADAM20 is a good candidate to participate in human gamete membrane adhesion and fusion, which is also supported by our finding that in the normal spermatozoa ADAM20 expressed in the head, especially in the putative acrosome region.

The D214A mutation is located in the pro-domain, which is not contained in the mature peptide of ADAM20. ADAM20 and ADAM21 were suggested to form a heterodimer complex. The mature complex was formed by cleavage of the pro-domain. Some ADAMs have the propeptide cleavage sites (RXKR and RRRR), while ADAM20 did not have an obvious cleavage site [3]. We speculated that some residues in the pro-domain under positive selection are of great importance for the normal function of ADAM20 and thus for sperm-egg fusion. A sequence (aa219-aa227) called “cysteine switch”, which is a short motif with an unpaired cysteine that is thought to interact with Zn^2+^ in the catalytic domain [8, 11], is near the D214 site. This switch suggested that ADAM20 requires proteolytic processing [8]. Therefore, we postulated that D214A may affect the function of proteolytic processing by interfering the interaction between cysteine switch motif and the catalytic domain. However, whether D214 site plays function in the heterodimerization, propeptide cleavage or secretion of the mature dimers needs to be further analyzed.

Mutations in ADAMs that have the dominant negative effects have been reported previously. EGFR transactivation induced by Ang II was inhibited in vascular smooth muscle cells infected by a dominant negative ADAM17 (E406A) [12]. The same dominant-negative ADAM17 adenovirus-treated carotid artery can markedly inhibit intimal hyperplasia [13]. Dominant negative ADMA10 (E384A) resulted in a decreased expression of ADAM10 [14]. So we hypothesized that the D214A mutation may possibly exert a dominant negative effect on ADAM20 function.

In conclusion, our study demonstrated for the first time that a mutation of *ADAM20*, may be associated with sperm-egg fusion disorder by affecting the localization of ADAM20 in the sperm head. However, this mutation did not affect early embryonic development and pregnant outcome if ICSI was carried out. Therefore, this study aids the IVF scientists to solve the sperm-egg fusion disorder and provides researchers with a new insight into the understanding the molecular mechanism of sperm-egg fusion process.

## MATERIALS AND METHODS

### Patient

The patient and his family were recruited from Xiamen Maternity and Child Care Hospital. This study was approved by the Ethics Committee of Xiamen Maternity and Child Care Hospital. Written informed consent was obtained and then 5 mL of peripheral blood was collected from each participant.

### Whole-exome sequencing (WES) analysis and validation by Sanger sequencing

WES was performed as previously described [15]. Full whole-exome sequencing data of the patient is available upon request. Sanger sequencing was used to validate the mutation of *ADAM20* in the proband, and in his mother and father.

### Immunostaining of spermatozoa

Immunostaining was performed as previously described [16]. The information of primary rabbit anti-ADAM20 antibody was listed in Supplementary Table 1.

## SUPPLEMENTARY MATERIALS FIGURES AND TABLES




