# Supplementary material for: Sperm-egg fusion disorder in a Chinese male patient was associated with a rare ADAM20 variant
Source: Oncotarget. 2017 Dec 16;9(2):2086–91. doi: 10.18632/oncotarget.23331 (PMC5788623; doi:10.18632/oncotarget.23331)
Supplement: Supplementary file 1 [file oncotarget-09-2086-s001.pdf]

Sperm-egg fusion disorder in a Chinese male patient was associated with a rare *ADAM20* variant

SUPPLEMENTARY MATERIALS

Supplementary Table 1: Antibodies used in Western Blot (WB) and Immunofluorescence (IF)

Primary/ Secondary antibodies	Source	Dilution	Cat no.
ADAM20	Rabbit polyclonal	1:1000 (WB)<br>1:100 (IF)	ABIN758831, antibodies-online
β-tubulin	Rabbit polyclonal	1:2000	10068-1-AP, Proteintech Group
Goat Anti-Rabbit IgG (H + L)		1:1000	ZB-2301, ZSGB-BIO

Supplementary Movie 1: The patient's spermatozoa can not fertilize the oocyte in the procedure of *in vitro* fertilization.
See Supplementary_Movie_1

A

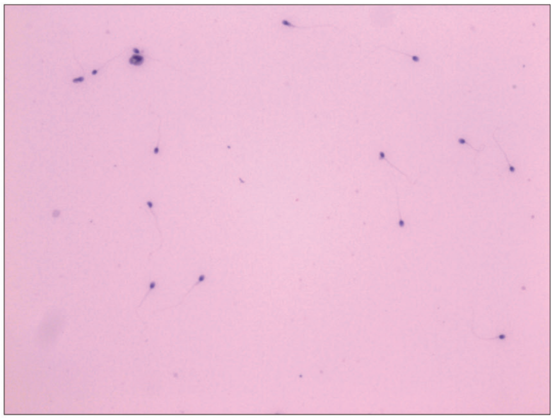

B

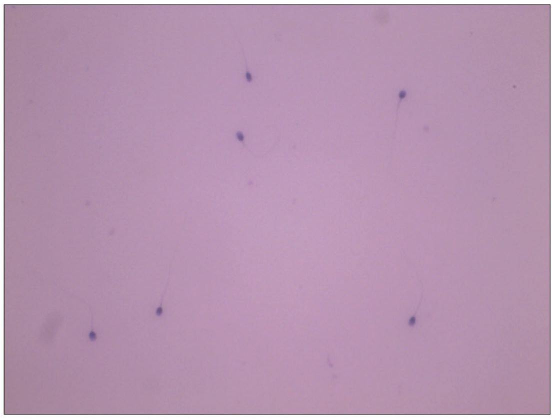

C

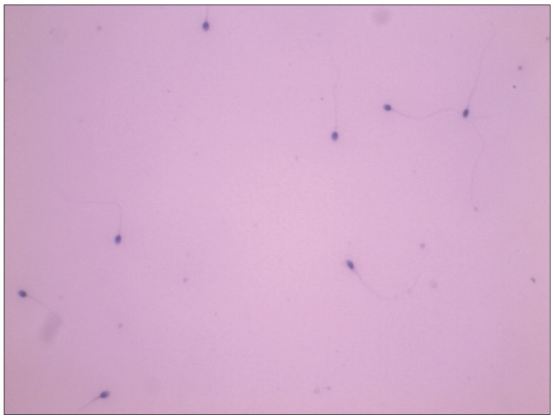

Supplementary Figure 1: (**A**–**C**) Papanicolaou staining of the patient's spermotozoa.
